# Supplementary material for: Liposomal Form of 2,4-Dinitrophenol Lipophilic Derivatives as a Promising Therapeutic Agent for ATP Synthesis Inhibition
Source: Nanomaterials (Basel). 2022 Jun 23;12(13):2162. doi: 10.3390/nano12132162 (PMC9268429; doi:10.3390/nano12132162)
Supplement: Supplementary file 1 [file nanomaterials-12-02162-s001.zip › nanomaterials-1741532-supplementary.pdf]

nanomaterials

Supplementary materials

Liposomal Form of 2,4-Dinitrophenol Lipophilic Derivatives as a Promising Therapeutic Agent for ATP Synthesis Inhibition

Vlasova K.Yu. [1,2], Ostroverkhov P.V. [3], Vedenyapina D.G. [3], Yakimova T.M. [4], Trusova A.B. [4], Lomakina G.Y. [2], Vodopyanov S.S. [5], Grin M.A. [3], Klyachko N.L. [2], Chekhonin V.P. [1,6] and Abakumov M.A. [1,5,6,*]

[1] Department of Medical Nanobiotechnology, Pirogov Russian National Research Medical University, 117997 Moscow, Russia; vlasova_k.y@mail.ru (K.Y.V.); chekhoninnew@yandex.ru (V.P.C.)
[2] School of Chemistry, Lomonosov Moscow State University, 119991 Moscow, Russia; lomakinagalina@yahoo.com (G.Y.L.); nlklyachko@gmail.com (N.L.K.)
[3] Department of Chemistry and Technology of Biologically Active Compounds, Medical and Organic Chemistry, Lomonosov Institute of Fine Chemical Technologies MIREA-Russian Technological University (RTU MIREA), 119571 Moscow, Russia; mrp_ost@mail.ru (P.V.O.); lykrecia@mail.ru (D.G.V.); grin@mirea.ru (M.A.G.)
[4] Faculty of Materials Science, Lomonosov Moscow State University, 119991 Moscow, Russia; yakimova_t31@inbox.ru (T.M.Y.); alla.trusova.fnm@gmail.com (A.B.T.)
[5] College of New Materials and Nanotechnologies, National University of Science and Technology (MISIS), 119049 Moscow, Russia; stepan.vodopianov@yandex.ru
[6] V. Serbsky National Medical Research Center for Psychiatry and Narcology, 119034 Moscow, Russia
* Correspondence: abakumov1988@gmail.com

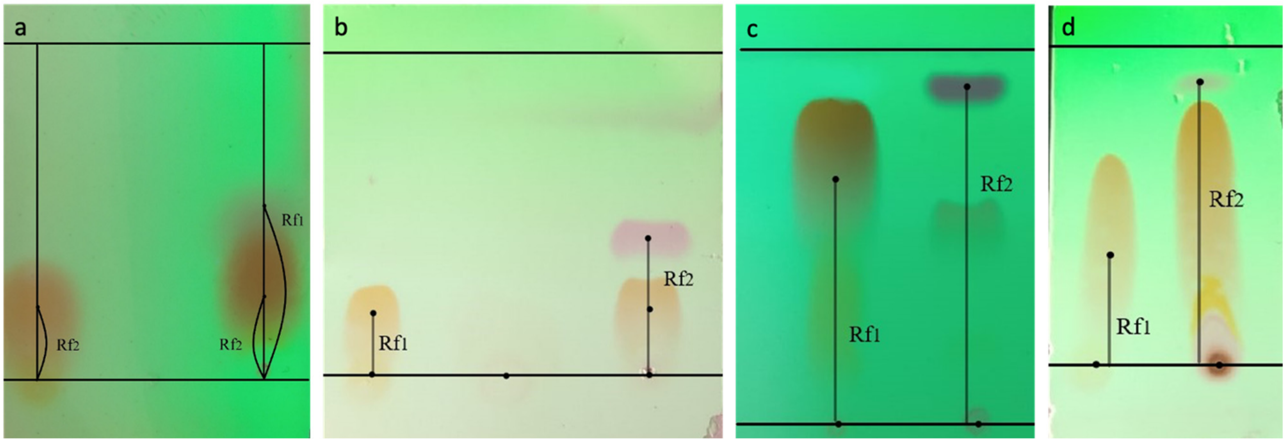

Figure S1. (**a**). TLC of 2,4-dinitrophenol(left) and reaction mixture for palmitic acid ester of 2,4-dinitrophenol via activated esters method. Eluent – hexane/chloroform = 1/1. Rf1 (ester) = 0.5; Rf2 (2,4-dinitrophenol) = 0.35; (**b**). TLC of 2,4-dinitrophenol(left) and reaction mixture for palmitic acid ester of 2,4-dinitrophenol via oxalyl chloride method. Eluent – pure hexane. Rf1 (2,4-dinitrophenol) = 0.6. Rf2 (ester) = 0.58; (**c**). TLC of 2,4-dinitrophenol(left) and reaction mixture for caproic acid ester of 2,4-dinitrophenol via oxalyl chloride method. Eluent – pure chloroform. Rf1 (2,4-dinitrophenol) = 0.66 Rf2 (ester) = 0.94; (**d**). TLC of 2,4-dinitrophenol(left) and reaction mixture for propanoic acid ester of 2,4-dinitrophenol via oxalyl chloride method. Eluent –hexane/chloroform = 2/1. Rf1 (2,4-dinitrophenol) = 0.375. Rf2 (ester) = 0.875

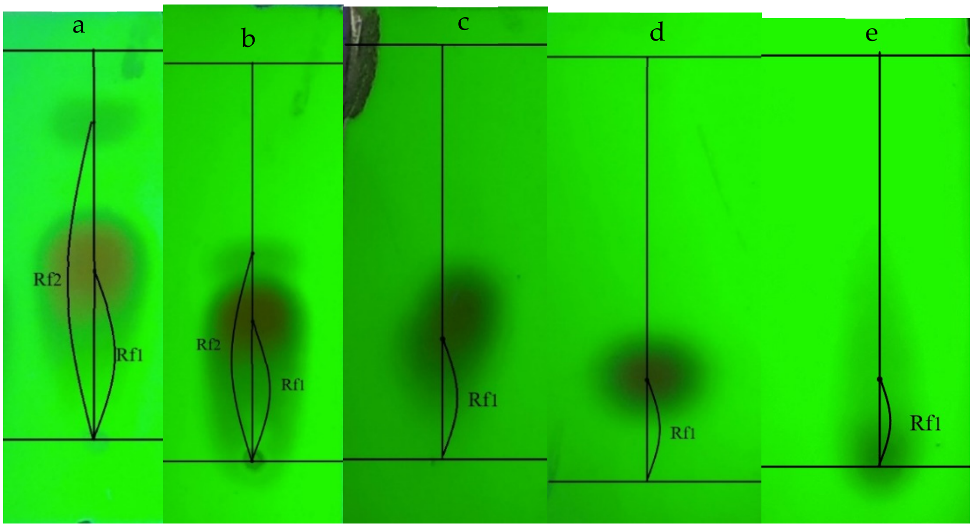

Figure S2. (**a**) TLC of reaction mixture for palmitic acid ester of 2,4-dinitrophenol via oxalyl chloride method. Eluent – CCl_4/methanol = 48/2. Rf1 (2,4-dinitrophenol) = 0.42. Rf2 (ester) = 0.83; (**b**). TLC of reaction mixture for palmitic acid ester of 2,4-dinitrophenol via oxalyl chloride method. Eluent – hexane/ethyl acetate = 48/2. Rf1 (2,4-dinitrophenol) = 0.33. Rf2 (ester) = 0.5; (**c**). TLC of reaction mixture for palmitic acid ester of 2,4-dinitrophenol via oxalyl chloride method. Eluent – pure benzene. Rf1 (mixture) = 0.33; (**d**). TLC of reaction mixture for palmitic acid ester of 2,4-dinitrophenol via oxalyl chloride method. Eluent – benzene/acetic acid = 80/20. Rf1 (mixture) = 0.25; (**e**). TLC of reaction mixture for palmitic acid ester of 2,4-dinitrophenol via oxalyl chloride method. Eluent – acetonitrile. Rf1 (mixture) = 0.31.

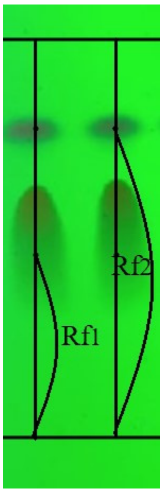

Figure S3. TLC of reaction mixture for palmitic acid ester of 2,4-dinitrophenol via oxalyl chloride method after first chromatographic column. Eluent – hexane/chloroform = 3/2.

Rf1 (2,4-dinitrophenol) = 0.42

Rf2 (palmitic ester) = 0.83

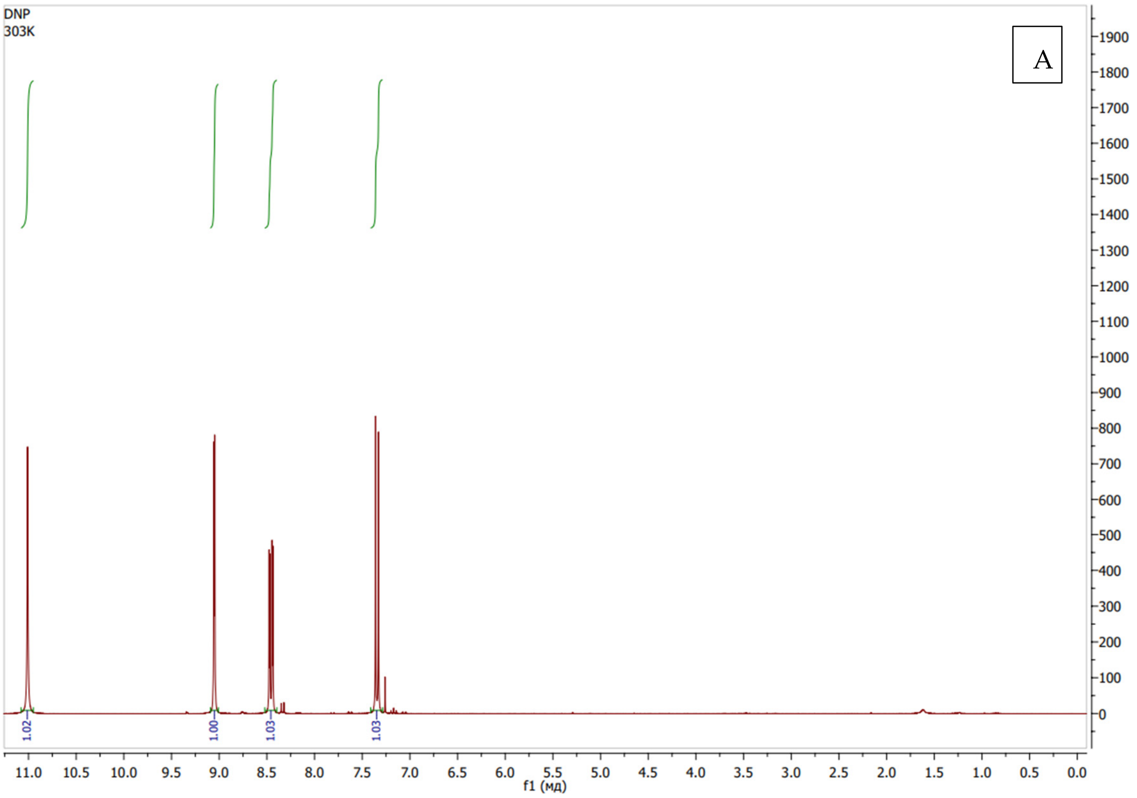
DNP
303K
A
1.02
1.00
1.03
1.03
11.0 10.5 10.0 9.5 9.0 8.5 8.0 7.5 7.0 6.5 6.0 5.5 5.0 4.5 4.0 3.5 3.0 2.5 2.0 1.5 1.0 0.5 0.0
f1 (мд)
1900 1800 1700 1600 1500 1400 1300 1200 1100 1000 900 800 700 600 500 400 300 200 100 0

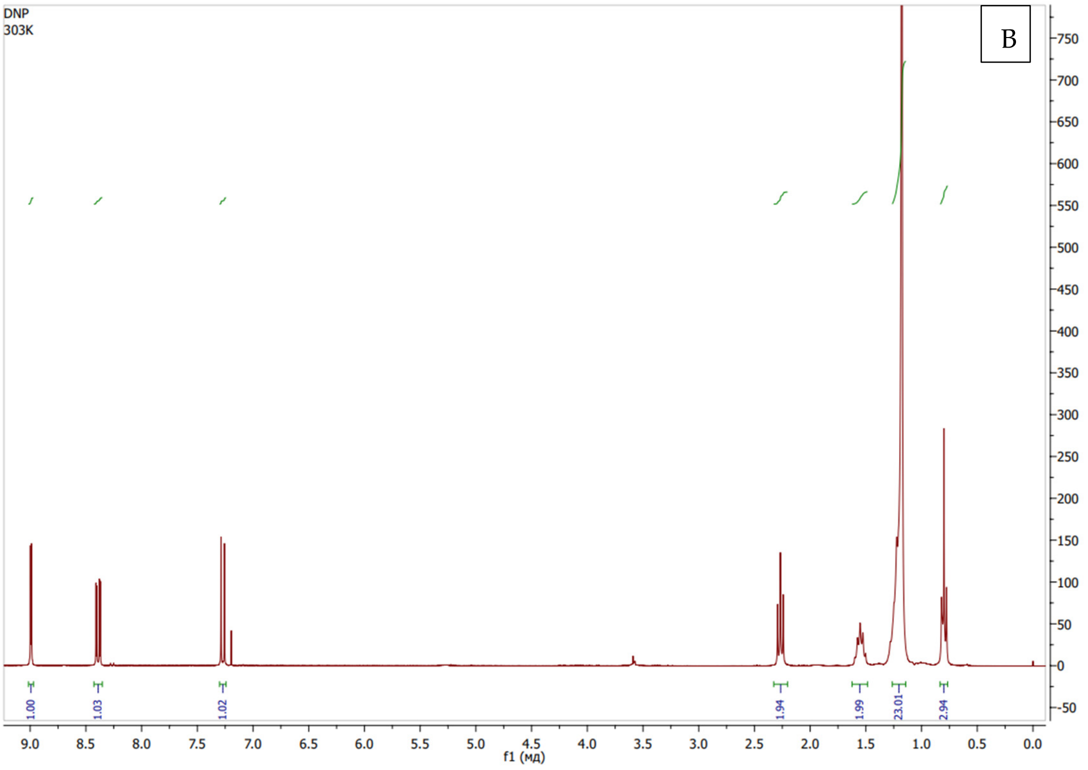
DNP
303K
B
1.00
1.03
1.02
1.94
1.99
23.01
2.94
9.0 8.5 8.0 7.5 7.0 6.5 6.0 5.5 5.0 4.5 4.0 3.5 3.0 2.5 2.0 1.5 1.0 0.5 0.0
f1 (мд)
750 700 650 600 550 500 450 400 350 300 250 200 150 100 50 0 -50

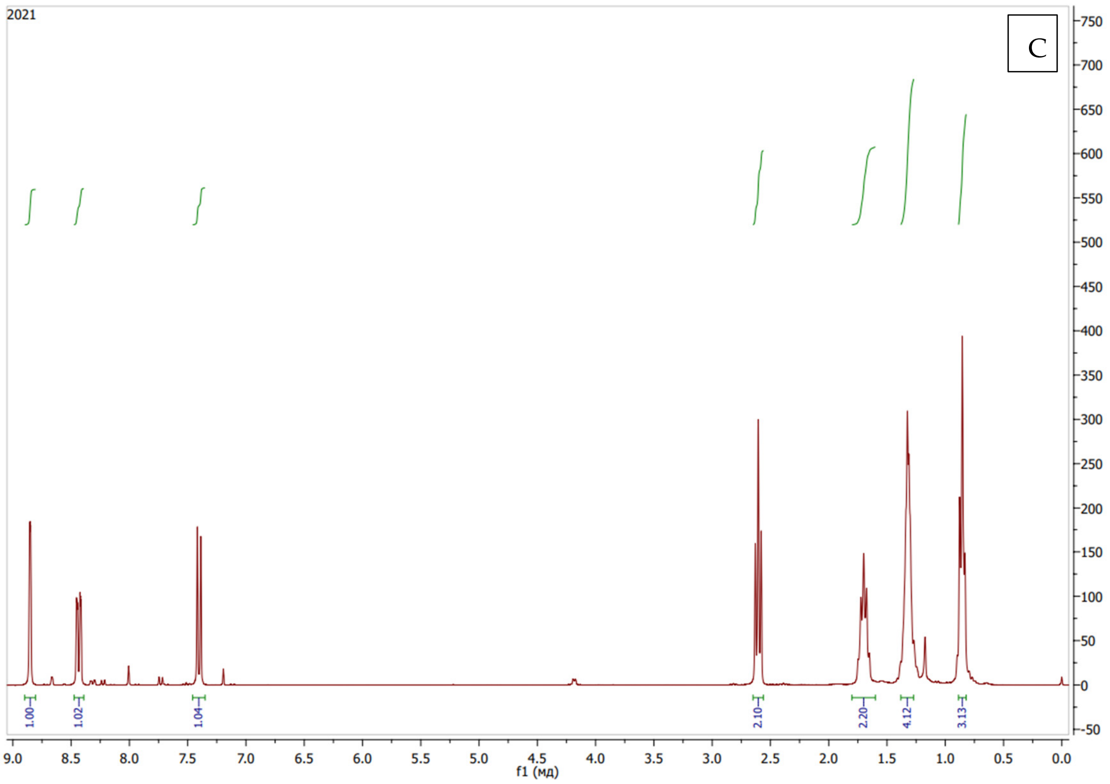

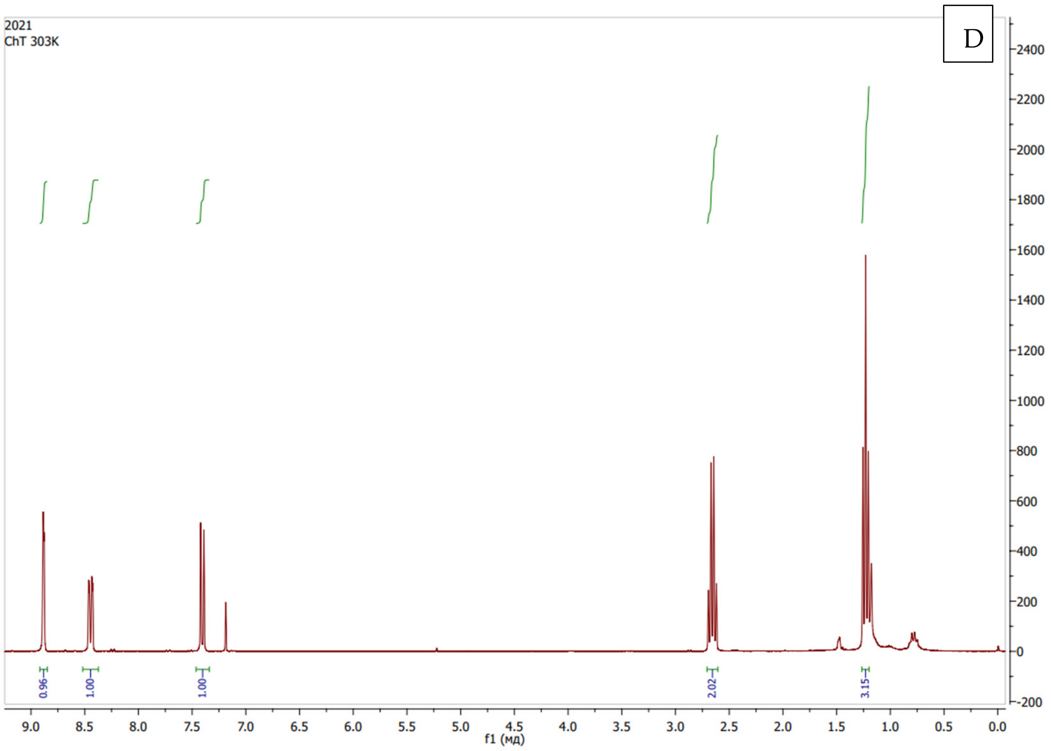

Figure S4. ^{1}H NMR spectra (300 MHz, $CDCl_3$): (**A**) compound 1, (**B**) compound 2, (**C**) compound 3, (**D**) compound 4.

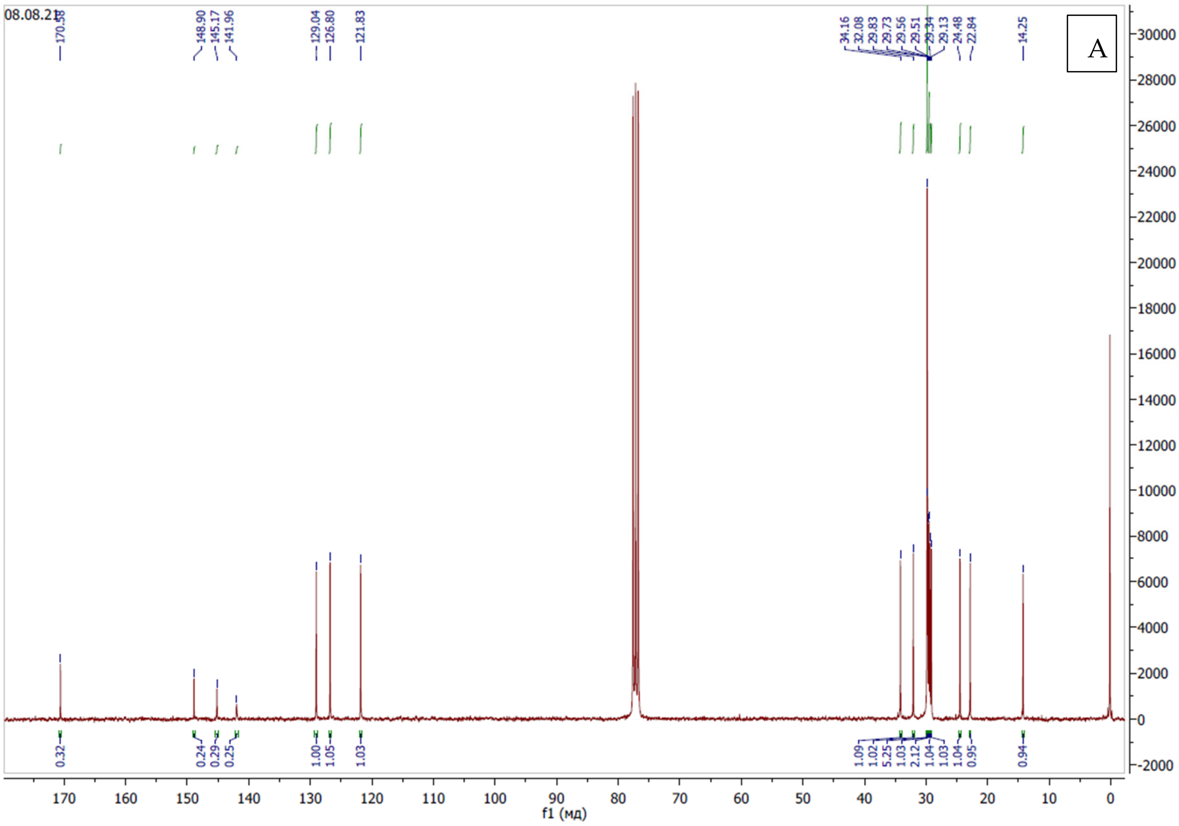
08.08.21
A
170.58
148.90
145.17
141.96
129.04
126.80
121.83
34.16
32.08
29.83
29.73
29.56
29.51
29.34
29.13
24.48
22.84
14.25
0.32
0.24
0.29
0.25
1.00
1.05
1.03
1.09
1.02
5.25
1.03
2.12
1.04
1.03
1.04
0.95
0.94
f1 (мд)

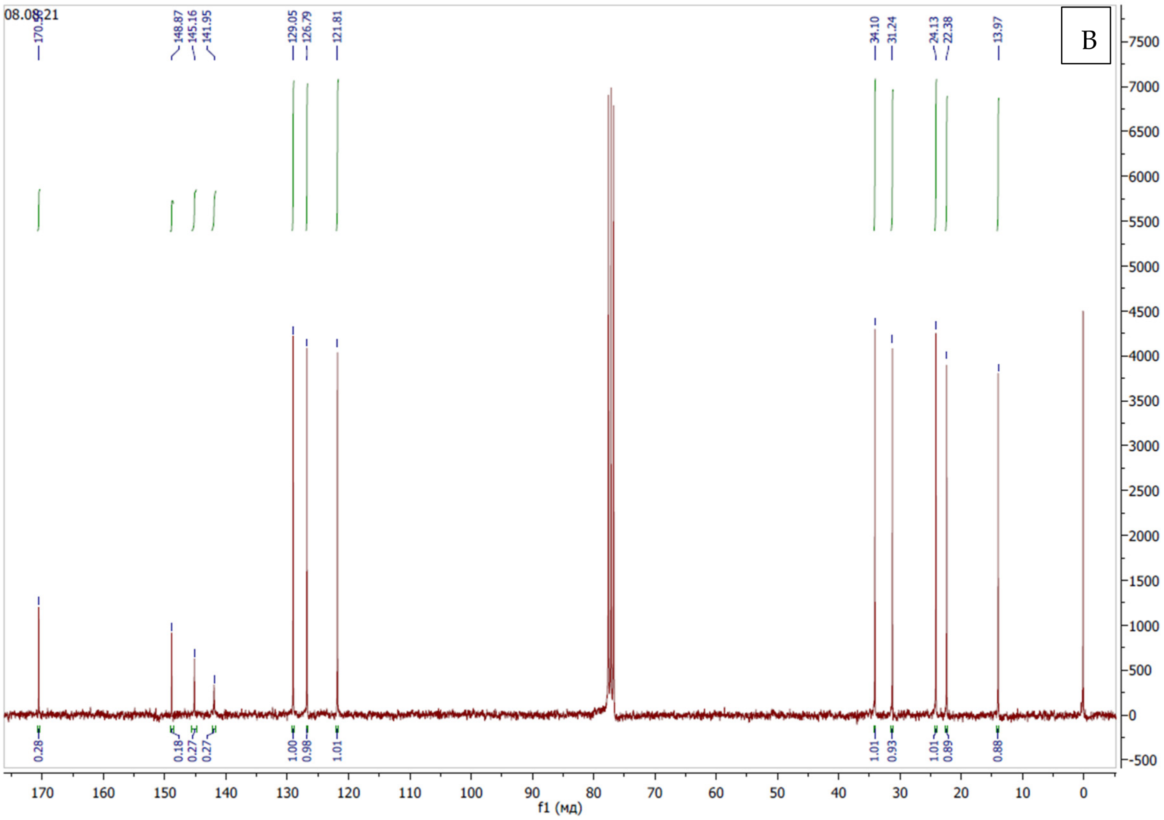
08.08.21
B
148.87
145.16
141.95
129.05
126.79
121.81
34.10
31.24
24.13
22.38
13.97
0.28
0.18
0.27
0.27
1.00
0.98
1.01
1.01
0.93
1.01
0.89
0.88
f1 (мд)

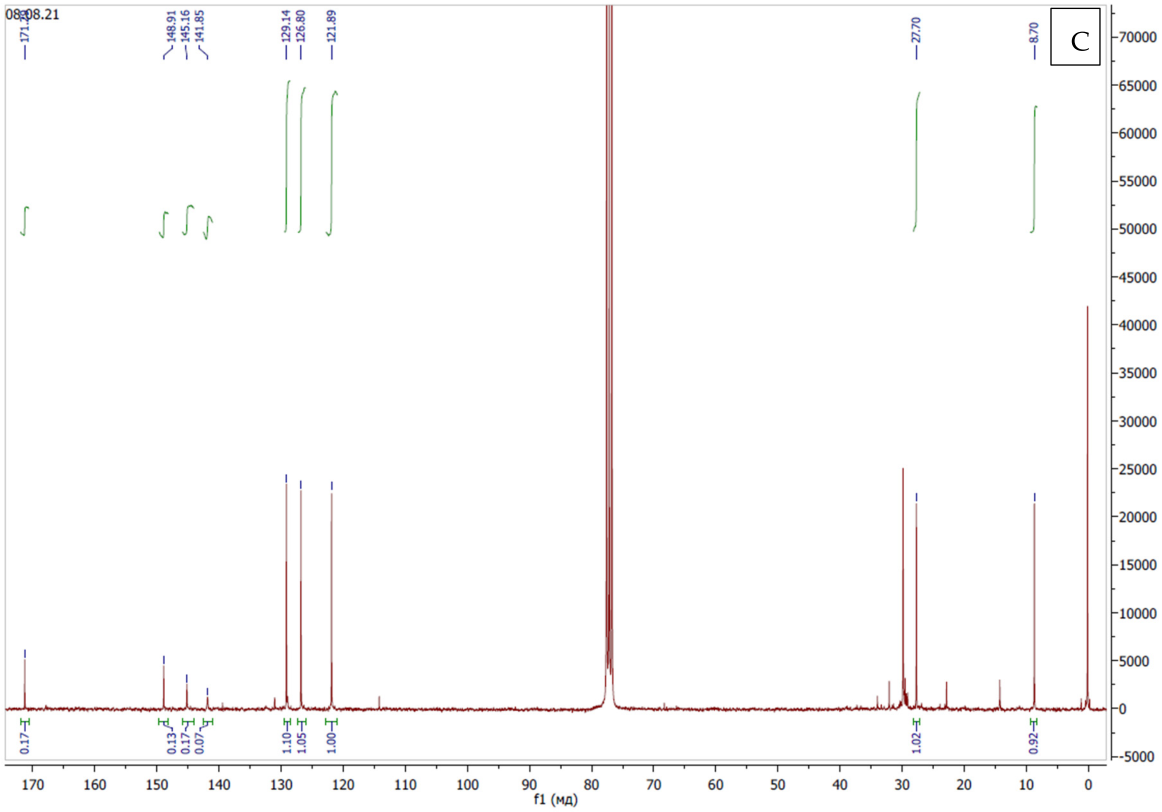

Figure S5. ^{13}C NMR spectra (300 MHz, $CDCl_3$): (**A**): compound 2, (**B**): compound 3, (**C**): compound 4.

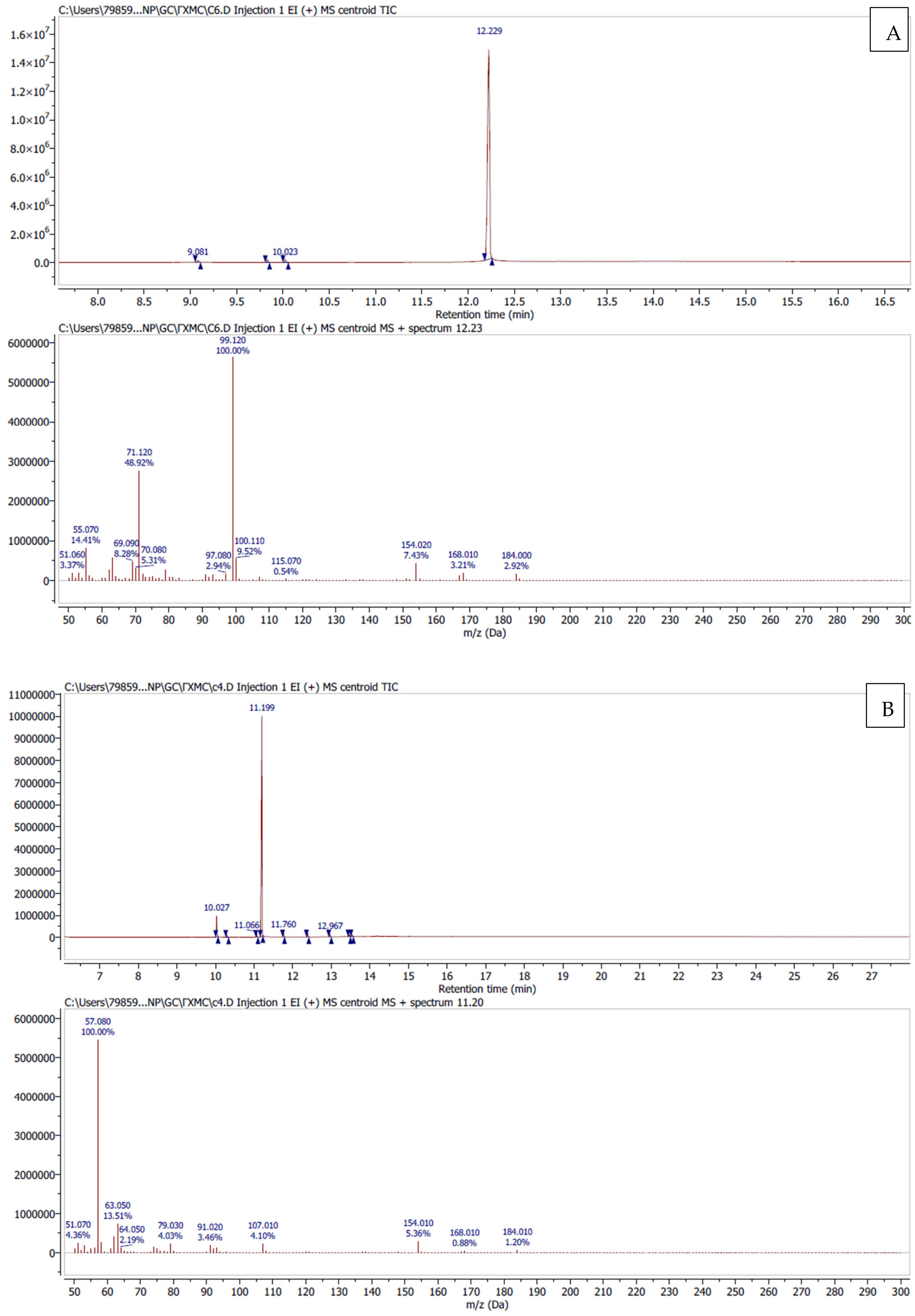

Figure S6. EI-MS and chromatogram: (**A**) compound 3, (**B**) compound 4.

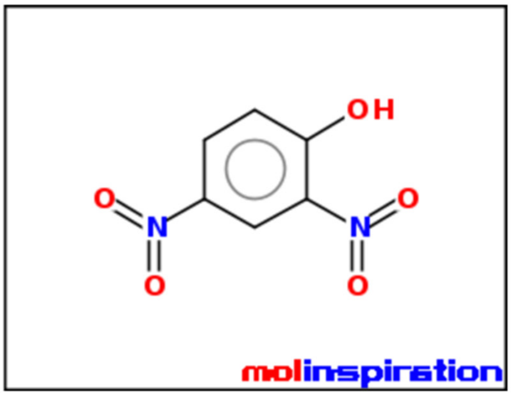

Molinspiration property engine v2018.10

miLogP	1.56
TPSA	111.88
natoms	13
MW	184.11
nON	7
nOHNH	1
nviolations	0
nrotb	2
volume	138.73

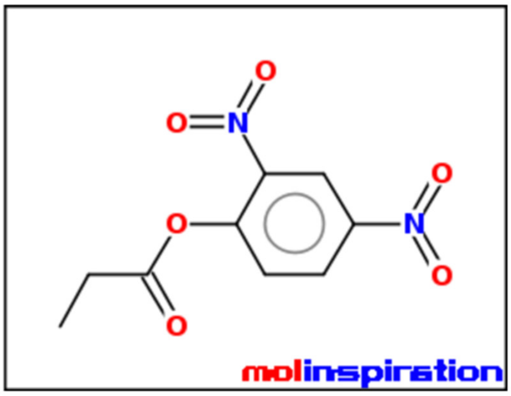

Molinspiration property engine v2018.10

miLogP	2.00
TPSA	117.95
natoms	17
MW	240.17
nON	8
nOHNH	0
nviolations	0
nrotb	5
volume	192.04

Get data as text (for copy / paste).

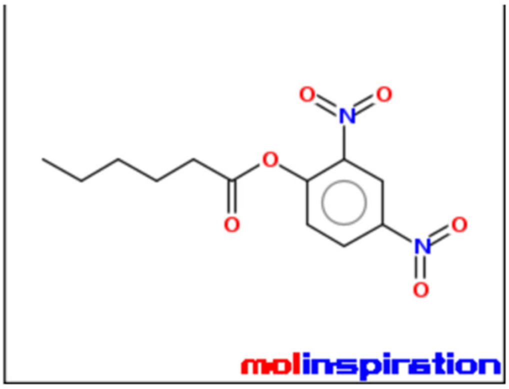

Molinspiration property engine v2018.10

miLogP	3.57
TPSA	117.95
natoms	20
MW	282.25
nON	8
nOHNH	0
nviolations	0
nrotb	8
volume	242.45

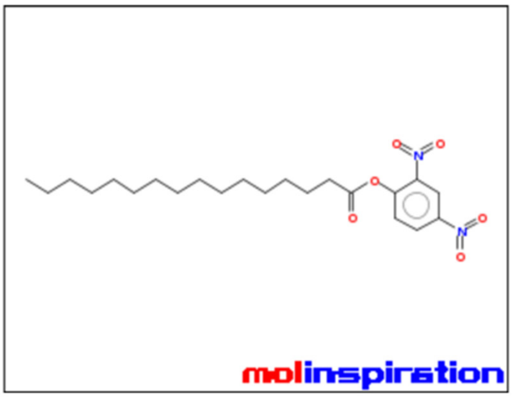

Molinspiration property engine v2018.10

miLogP	8.49
TPSA	117.95
natoms	30
MW	422.52
nON	8
nOHNH	0
nviolations	1
nrotb	18
volume	410.47

Get data as text (for copy / paste).

Figure S7. Theoretically estimated $LogP_{oct}$ of the synthesized molecules.

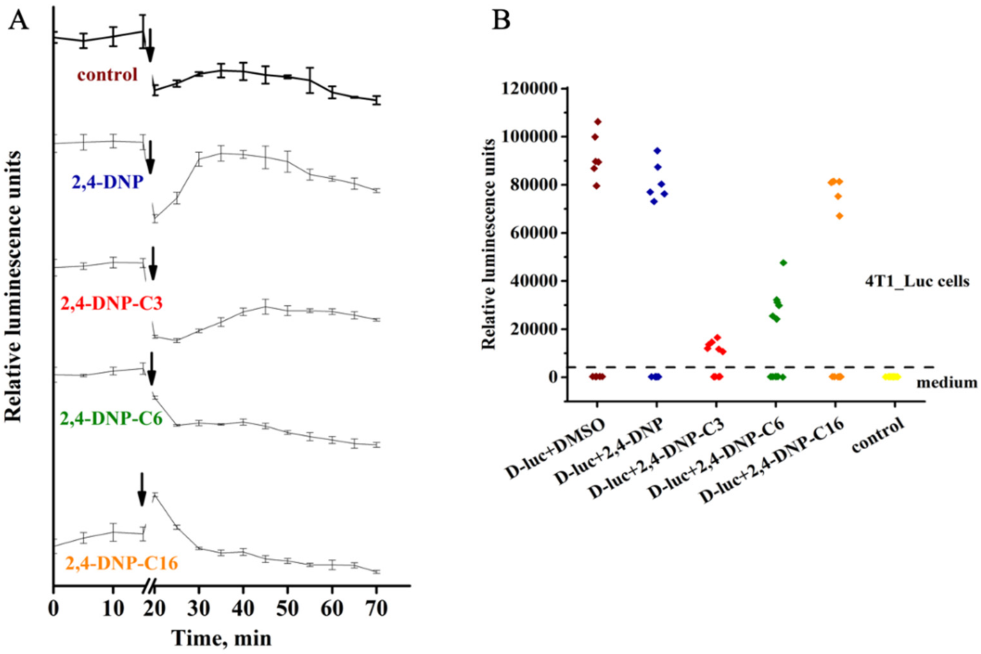

Figure S8. Luciferin-luciferase coupled assay using 4T1-Luc cell line in RPMI 1640 (10% FBS) at 37 °C. The total luciferin concentration was 150 µg/ml; 2,4-DNP (compound **1**), 2,4-DNP-C16 (compound **2**), 2,4-DNP-C6 (compound **3**), 2,4-DNP-C3 (compound **4**) concentrations were 200 µM per well. Samples were dissolved in DMSO. The final concentration of DMSO was 1% per well. (**A**) Luminescence signal of the reaction before and after addition of the tested compounds. Arrows indicate the compound addition point. The sample with untreated cells was set as control. Data are presented as Mean ± StD (n = 3). (**B**) Luminescence signal of the reaction in cells and external medium after 2 h incubation of the cells with D-luciferin+DMSO, D-luciferin+2,4-DNP, D-luciferin+2,4-DNP-C16, D-luciferin+2,4-DNP-C6, D-luciferin+2,4-DNP-C3. The cells in medium without D-luciferin were set as control. (n = 6). D-luciferin was added to the cells and external medium after treatment (the final concentration 150 µg/ml) and the mixture was incubated for 10 min. The average cells concentration was $2*10^5$ per well.

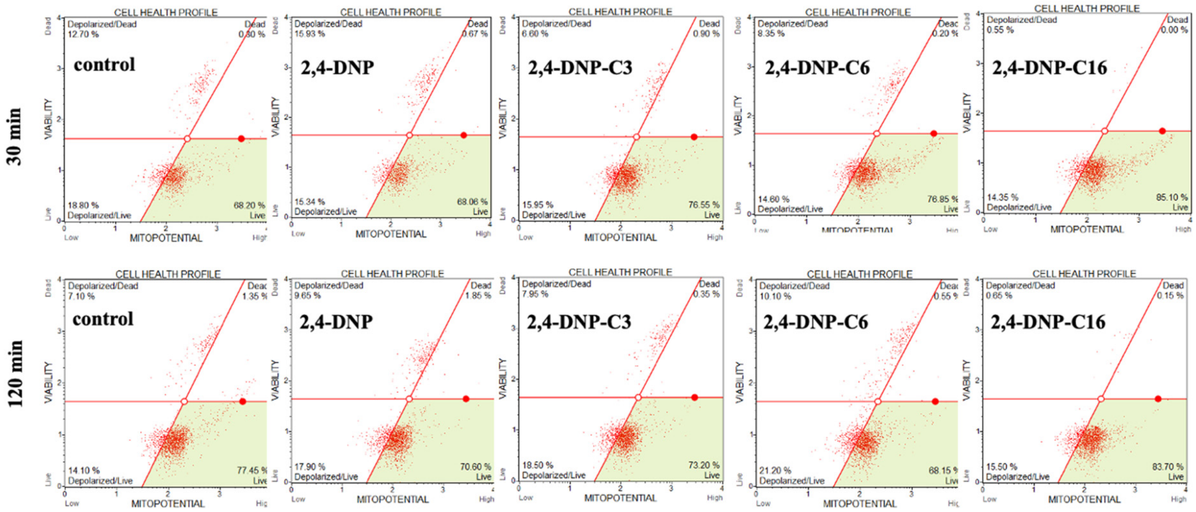

Figure S9. Change in mitochondrial potential after 2,4-DNP (compound **1**), 2,4-DNP-C16 (compound **2**), 2,4-DNP-C6 (compound **3**), 2,4-DNP-C3 (compound **4**) cell treatments. 4T1-Luc cells were incubated with the tested compounds (200 µM) for 30 min and 120 min in RPMI 1640 (10% FBS) at 37 °C, 5% CO_2, then the mitochondrial potential was assessed by a cytofluorimetric technique. Samples with untreated cells were set as control. Representative cytofluorimetric population profiles.

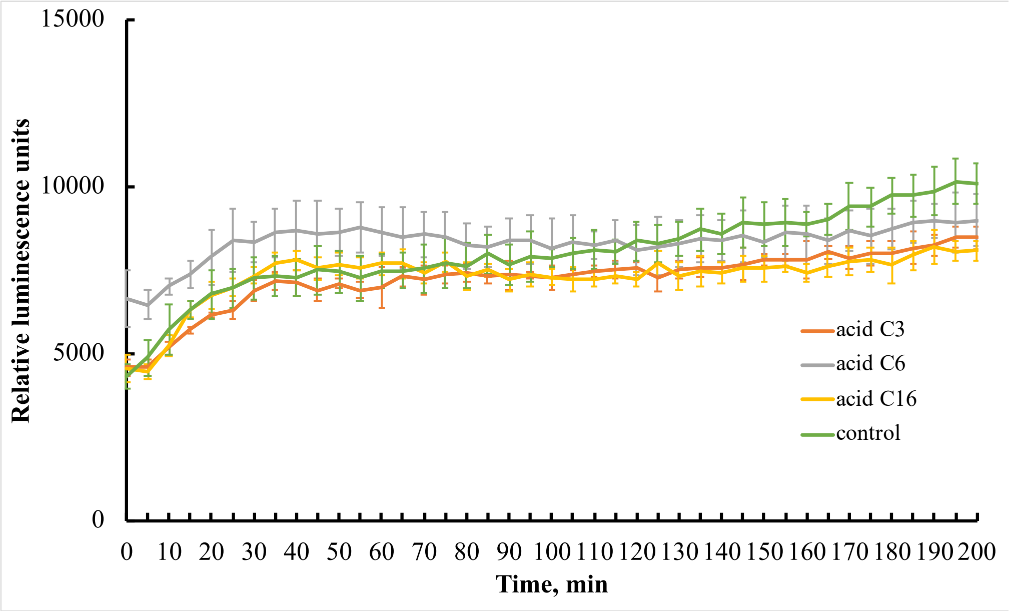

Figure S10. Luciferin-luciferase coupled assay kinetics using 4T1-Luc cell line in RPMI 1640 (10% FBS) at 37 °C. The total luciferin concentration was 150 µg/ml; propionic (C3), caproic (C6) and palmitic (C16) acids concentrations were 200 µM. Acids were dissolved in DMSO before the experiment. The total concentration of DMSO in final solution was 1%. Samples with untreated cells (only luciferin addition) were set as control. Data are presented as Mean±StD (n = 3).

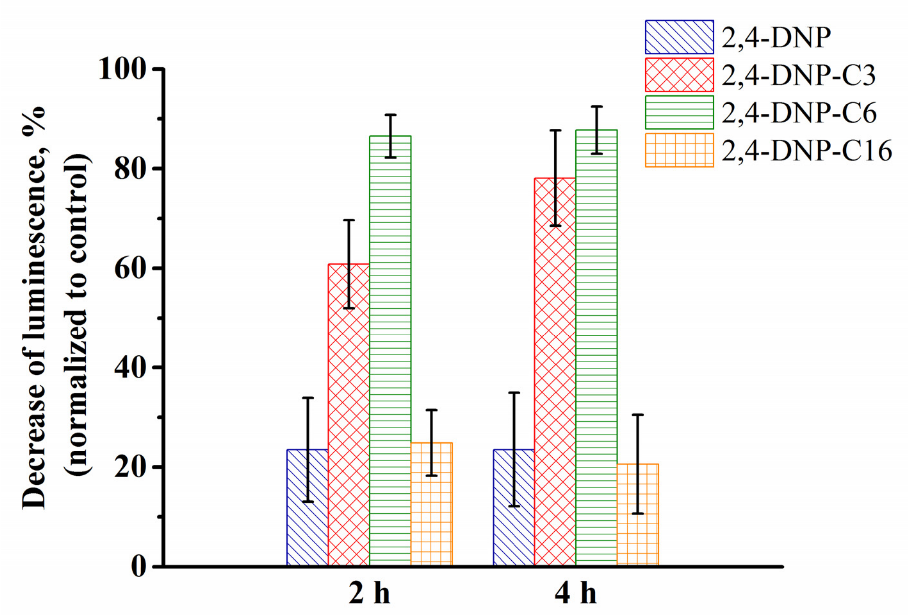

Figure S11. Luciferin-luciferase coupled assay using 4T1-Luc cell line in RPMI 1640 (10% FBS) at 37 °C, 5% CO_2. The total D-luciferin concentration was 150 µg/ml; 2,4-DNP (compound **1**), 2,4-DNP-C16 (compound **2**), 2,4-DNP-C6 (compound **3**), 2,4-DNP-C3 (compound **4**) concentrations were 200 µM per well. Samples were dissolved in DMSO. The final concentration of DMSO was 1% per well. Decrease of luminescence signal after addition of the D-luciferin (150 µg/ml) to the treated with the tested compound cells normalized to the untreated cells (control). Data are presented as Mean±StD (n = 6). The average cell concentration was 2.3*10^5 cell/ml.

Procedure: 4T1-Luc cells were seeded in black 96-well plates (Greiner, France) (1×10^4 cells/well) 48 h before the experiment. Cell medium was replaced with the mixtures of medium alone (control) and with examined substance (200 µM of free 2,4-DNP or its derivatives) in RPMI 1640 medium (10% FBS, 4.5 g/L glucose, 100 U/ml penicillin, 100 µg/ml streptomycin и 0.25 µg/ml Gibco amphotericin). After 2 h or 4 h incubation the medium

was replaced with the mixture of D-luciferin (150 μg/ml). After 10 min of incubation the plate was put into the Luminometer chamber (EnSpire multimode plate reader, Perkin Elmer, USA) and the luminescence signal was detected at 37 °C (flashes/time 0.1 sec). 2,4-DNP esters were dissolved in DMSO before the use. The total concentration of DMSO in final solution per well was 1%.

The inhibition effect was expressed as

$$Decrease\ of\ luminescence\ =\ \frac{[RLU]control-[RLU]}{[RLU]control}*100\%'$$

where $[RLU]_{control}$ – relative luminescence units of the untreated cells (control); [RLU] – relative luminescence units at certain time point of the treated cells.

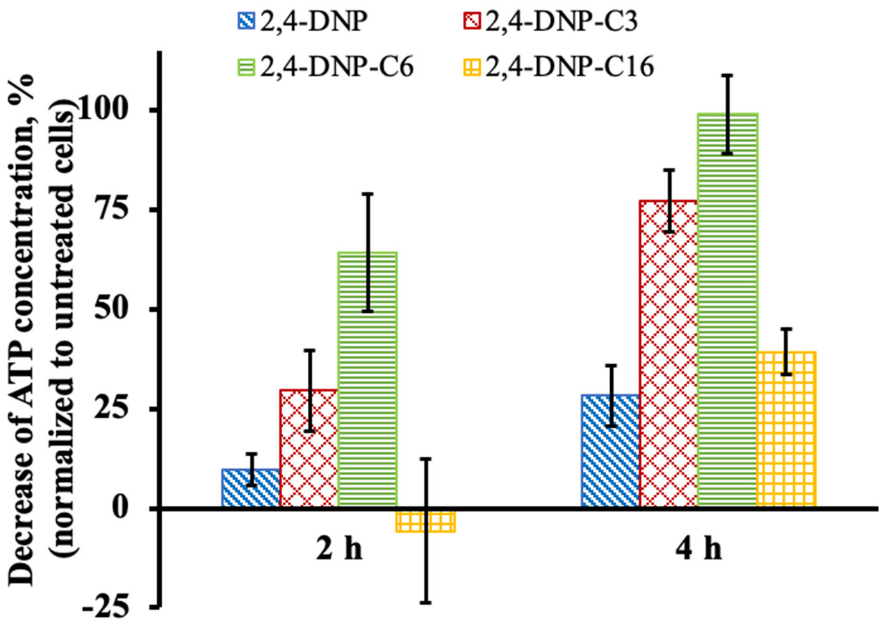

Figure S12. Decrease of ATP content in 4T1-Luc cells after treating with the 200 μM 2,4-DNP (compound **1**), 2,4-DNP-C16 (compound **2**), 2,4-DNP-C6 (compound **3**), 2,4-DNP-C3 (compound **4**) for 2 h and 4 h, normalized to the ATP content in 4T1_Luc untreated cells. Samples were dissolved in DMSO. The final concentration of DMSO was 1% per well. Data are presented as Mean±StD (n = 2-3). The average cell concentration was $1.25*10^5$ cell/ml.

Procedure: 4T1_Luc cells were seeded into 96-well plate in 48 h before the experiment. Cell medium was replaced with the mixtures of medium alone (control) and with examined substance (200 μM of free 2,4-DNP or its derivatives) in RPMI 1640 medium (10% FBS, 4.5 g/L glucose, 100 U/ml penicillin, 100 μg/ml streptomycin и 0.25 μg/ml Gibco amphotericin). Samples were dissolved in DMSO. The final concentration of DMSO was 1% per well. After 2 h or 4 h incubation the total ATP content in the medium and cells was determined as follows: a 20 μL sample of the cell suspension was taken into a test tube, and 180 μL of DMSO was added. After 1 min incubation, 20 μL of the obtained extract was taken into a polystyrene microcuvette (cat. N507050, Grenier, France), 100 μL of ATP-reagent (the mixture of luciferase, D-luciferin, $MgSO_4$ and buffer) was added and bioluminescence signal was detected (for 30 s) using luminometer FB-12 (Berthold Detection Systems GmbH, Pforzheim, Germany). The mean value of the signal $I_{extract}$ was calculated. The bioluminescence signal was measured in a similar manner in the ATP control solution ($I_{control}$). The ATP concentration (ATP_{tot}) was calculated using the formula 1:

$$[ATPtot]\ =\ 10*[ATPcontrol]\frac{Iextract}{Icontrol},$$

where [$ATP_{control}$] = 3.75 nM in 90% DMSO, coefficient 10 is a dilution coefficient. The results were expressed as

$$Decrease\ of\ ATP\ concentration\ =\ \frac{[ATP]control-[ATP]}{[ATP]control} * 100\%'$$

where $[ATP]_{control}$ – ATP content in the untreated cells (control); [ATP] – ATP contetn at certain time point in the treated cells.

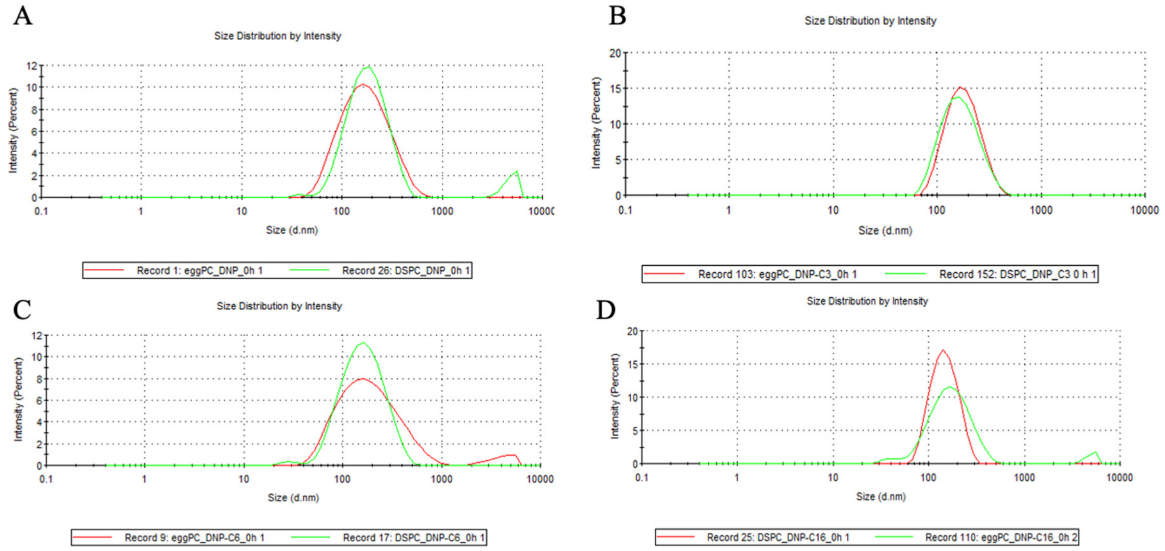

Figure S13. Size distribution of (**A**) 2,4-DNP loaded into eggPC (red line) and DSPC (green line) liposomes; (**B**) 2,4-DNP-C3 loaded into eggPC (red line) and DSPC (green line) liposomes; (**C**) 2,4-DNP-C6 loaded into eggPC (red line) and DSPC (green line) liposomes; (**D**) 2,4-DNP-C16 loaded into eggPC (green line) and DSPC (red line) liposomes in 10 mM PBS (pH 7.4).

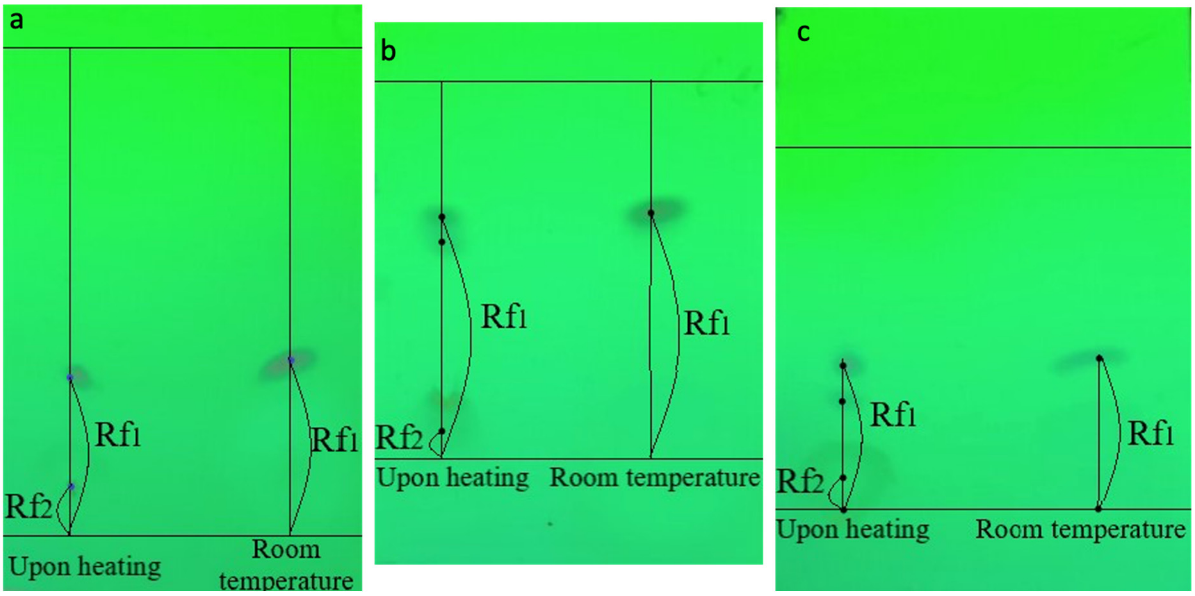

Figure S14. Stability of (**a**) 2,4-DNP-C16 (compound 2), (**b**) 2,4-DNP-C6 (compound 3) and (**c**) 2,4-DNP-C3 (compound 4) in PBS (pH = 7,4) with and without heating up to 65 °C.

Procedure: All samples were dispersed in 10 mM PBS (pH = 7.4). Then the aliquots were incubated at a room temperature and at 65 °C for 1 h. After that we made several TLC for each sample representing heated and non-heated substances. It can be clearly seen that after 1 h upon heating all the esters were partly decomposed, though there was still a strong presence of all the esters even after such rather harsh conditions. Samples incubated at a room temperature did not experience any visible changes.

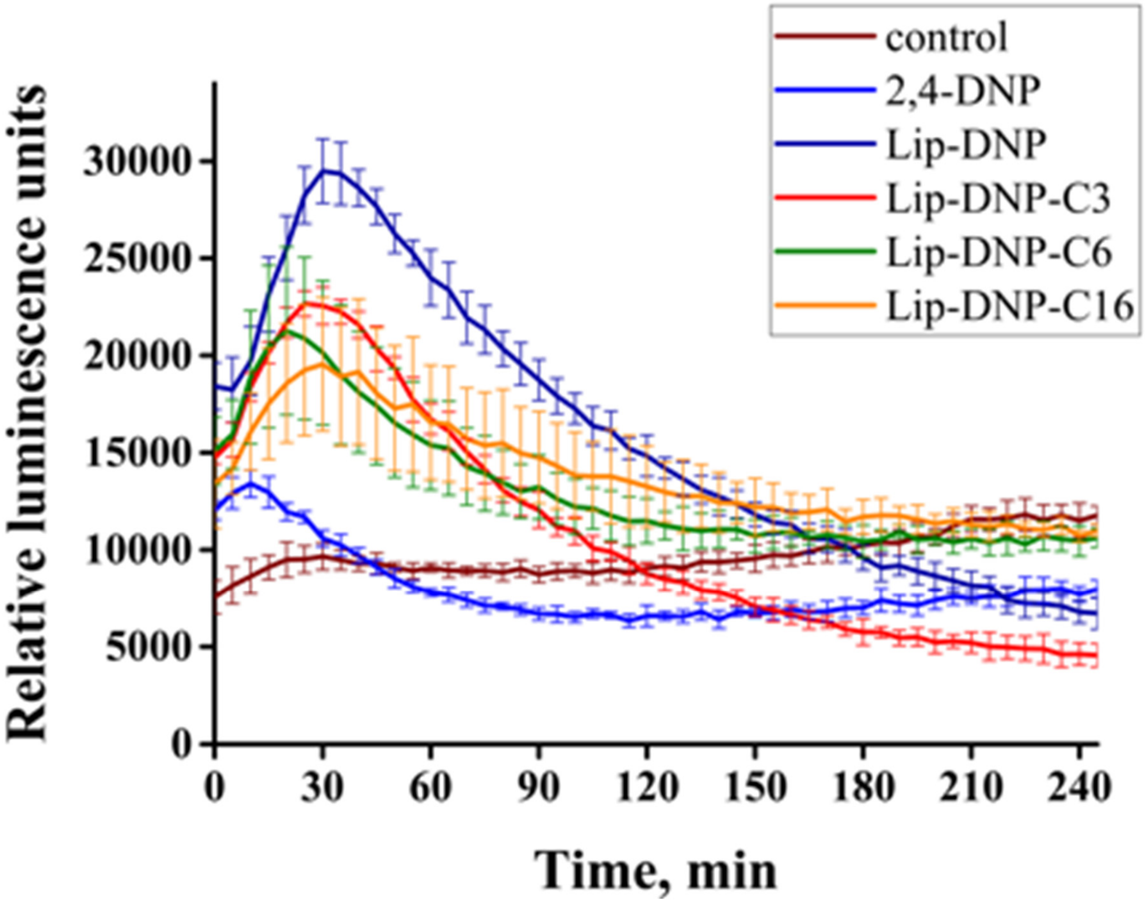

Figure S15. Luciferin-luciferase coupled assay for eggPC liposomes loaded with 2,4-DNP (compound **1**), 2,4-DNP-C16 (compound **2**), 2,4-DNP-C6 (compound **3**), 2,4-DNP-C3 (compound **4**). The total luciferin concentration was 150 μg/ml; 2,4-DNP (compound **1**), 2,4-DNP-C16 (compound **2**), 2,4-DNP-C6 (compound **3**), 2,4-DNP-C3 (compound **4**) concentrations were 200 μM per well. Samples with untreated cells (only luciferin addition) were set as control. Test was provided using 4T1-Luc cell line in RPMI 1640 (10% FBS) at 37 °C. Data are presented as Mean±StD (n = 6).

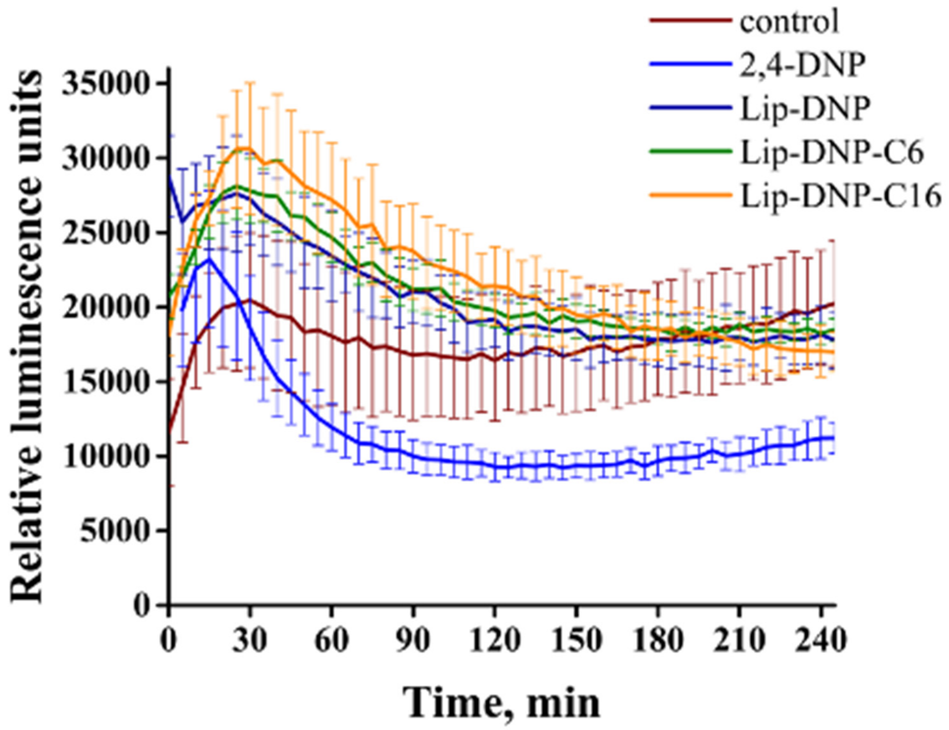

Figure S16. Luciferin-luciferase coupled assay for DSPC liposomes loaded with 2,4-DNP (compound **1**), 2,4-DNP-C16 (compound **2**), 2,4-DNP-C6 (compound **3**). The total luciferin concentration was 150 µg/ml; 2,4-DNP (compound **1**), 2,4-DNP-C16 (compound **2**), 2,4-DNP-C6 (compound **3**) concentrations were 200 µM per well. Samples with untreated cells (only luciferin addition) were set as control. Test was provided using 4T1-Luc cell line in RPMI 1640 (10% FBS) at 37 °C. Data are presented as Mean±StD (n = 5).

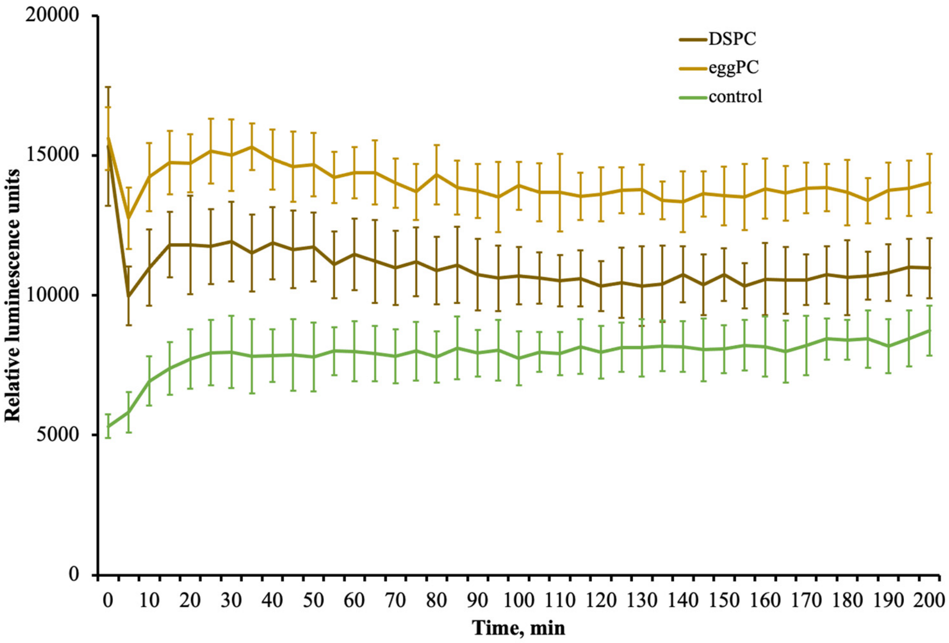

Figure S17. Luciferin-luciferase coupled assay kinetics using 4T1-Luc cell line in RPMI 1640 (10% FBS) at 37 °C after addition of eggPC and DSPC liposomes. The total luciferin concentration was 150 µg/ml; the lipid concentration was 12 mg/ml, that is refer to the maximal concentration used in experiments. Samples with untreated cells (only luciferin addition) were set as control. Data are presented as Mean±StD (n = 6).

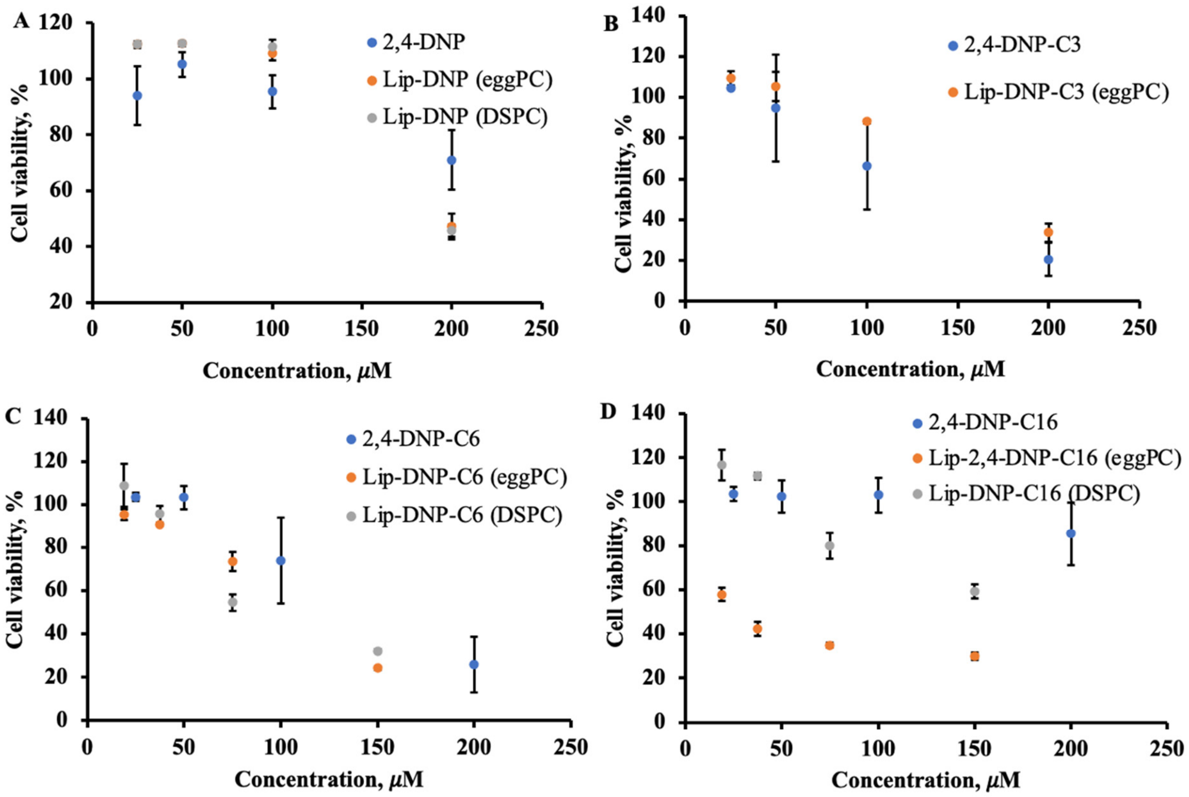

Figure S18. Cytotoxicity of free and encapsulated into liposomes, (**A–D**): 2,4-DNP (compound 1), 2,4-DNP-C16 (compound 2), 2,4-DNP-C6 (compound 3) and 2,4-DNP-C3 (compound 4) on 4T1-Luc cells after 24 h incubation in RPMI 1640 (10% FBS) at 37 °C, 5% CO_2. Untreated cells were set as a control. The free compounds were dissolved in DMSO. The final concentration of DMSO was 1% per well. Data are presented as Mean ± StD (n = 3–5).

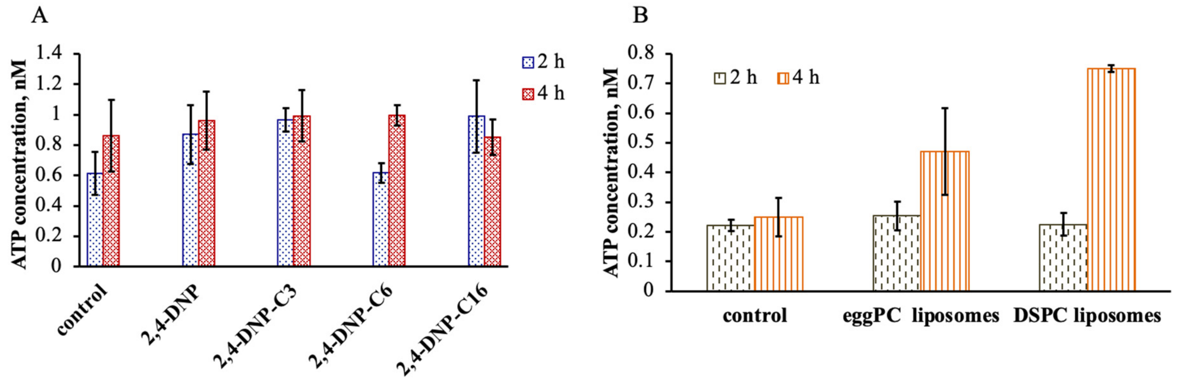

Figure S19. ATP content in AML12 cells (**A**) after treating with the 200 µM 2,4-DNP (compound **1**), 2,4-DNP-C16 (compound **2**), 2,4-DNP-C6 (compound **3**), 2,4-DNP-C3 (compound **4**) for 2 h and 4 h. Samples were dissolved in DMSO. The final concentration of DMSO was 1% per well; (**B**) after addition of empty eggPC and DSPC liposomes. Data are presented as Mean±StD (n = 3–4). The average cell concentration was 1*10^4 cell/ml.

Procedure: AML12 cells were seeded into 96-well plate in 48 h before the experiment. Cell medium was replaced with the mixtures of medium alone (control) and with examined substance (200 µM of free 2,4-DNP or its derivatives) in DMEM-F12 medium (10% FBS, 4.5 g/L glucose, 100 U/ml penicillin, 100 µg/ml streptomycin и 0.25 µg/ml Gibco amphotericin). Samples were dissolved in DMSO. The final concentration of DMSO was 1% per well. After 2 h or 4 h incubation the total ATP content in the medium and cells was determined as follows: a 20 µL sample of the cell

suspension was taken into a test tube, and 180 μL of DMSO was added. After 1 min incubation, 20 μL of the obtained extract was taken into a polystyrene microcuvette (cat. N507050, Grenier, France), 100 μL of ATP-reagent (the mixture of luciferase, D-luciferin, $MgSO_4$ and buffer) was added and bioluminescence signal was detected (for 30 s) using luminometer FB-12 (Berthold Detection Systems GmbH, Pforzheim, Germany). The mean value of the signal $I_{extract}$ was calculated. The bioluminescence signal was measured in a similar manner in the ATP control solution ($I_{control}$). The ATP concentration (ATP_{tot}) was calculated using the formula 1:

$$[ATPtot] = 10 * [ATPcontrol] \frac{Iextract}{Icontrol},$$

where [$ATP_{control}$] = 3.75 nM in 90% DMSO, coefficient 10 is a dilution coefficient.
